# Skin prick testing in patients using beta-blockers: a retrospective analysis

**DOI:** 10.1186/1710-1492-6-2

**Published:** 2010-01-20

**Authors:** Irene N Fung, Harold L Kim

**Affiliations:** 1 McMaster University, Hamilton, Ontario, Canada; 2 University of Western Ontario, London, Ontario, Canada

## Abstract

**Rationale:**

The use of beta-blockers is a relative contraindication in allergen skin testing yet there is a paucity of literature on adverse events in this circumstance. We examined a population of skin tested patients on beta-blockers to look for any adverse effects.

**Methods:**

Charts from 2004-2008 in a single allergy clinic were reviewed for any patients taking a beta-blocker when skin tested. Data was examined for skin test reactivity, type of skin test, concomitant asthma diagnosis, allergens tested, and adverse events.

**Results:**

One hundred and ninety-one patients were taking beta-blockers when skin testing occurred. Seventy-two patients had positive skin tests. No tests resulted in an adverse event.

**Conclusions:**

This data demonstrates the relative safety of administrating of skin prick tests to patients on beta-blocker treatment. Larger prospective studies are needed to substantiate the findings of this study.

## Introduction

Beta antagonists, commonly known as beta-blockers, are a commonly prescribed class of medications. Beta-blockers are used in the treatment of congestive heart failure, coronary heart disease, cardiac arrhythmia, hypertension, tremor, glaucoma, and migraine headache. Importantly, beta-blockers significantly reduce both morbidity and mortality rates in congestive heart failure, in acute coronary syndrome, and post myocardial infarction [1-3]. However, beta-blockade may place atopic subjects at an increased risk of an anaphylactic reaction. Case reports suggest that when systemic allergic reactions occur secondary to immunotherapy, drugs, foods, and insects stings, they may be of greater severity in patients taking beta-blockers [4-11].

Due to the potential of beta-blockers to amplify the effects of anaphylaxis, these drugs are relatively contraindicated during allergy skin testing. The American Academy of Allergy Asthma & Immunology (AAAAI) outlines this in its position statement, stating that "Systemic reactions to skin testing are rare. Nevertheless, special precautions, when these are appropriate, should be taken when the patient who needs sensitivity testing for IgE-mediated disease cannot stop treatment with a beta-blocking agent [12]." However, in our literature review on the topic, no case reports or prospective studies report adverse events in patients on beta-blockers who underwent skin testing. This retrospective study investigates whether there is any increased risk of anaphylaxis in patients who were allergy skin tested while they continued on a beta-blocker medication.

## Methods

Charts of all patients seen at an allergy clinic in Kitchener, Ontario from 2004 and 2008 were searched for any beta-blocker use at the time of their clinic visit. These records were examined to identify if any individuals who were skin tested, while they continued to take beta-blockers, had any reactions to the skin testing. The charts of the patients who were found to be allergic, based on skin testing, were further analysed regarding the medical history, type of beta-blocker taken and skin test findings.

## Results

One hundred and ninety-one patients were identified to be taking beta-blockers while undergoing skin testing. From the review of their charts, none of these patients experienced any adverse event secondary to skin testing. Specifically, there were no cases of anaphylaxis in these patients. Also, all patients who were taking beta-blockers at the time of the allergy consultation were skin tested.

Seventy-two patients on beta-blockers had positive results on skin testing, thus demonstrating an IgE-mediated sensitivity. The mean age of these patients was 60 (range 34-89), and 58% of these patients were male. Some other demographic data for the patients with positive skin test results is reported in Table 1. A few patients had more than one primary reason for referral. Spirometry findings and concomitant medications are also presented in the table as these two factors could have affected the severity of an anaphylactic reaction. Fifteen of the 72 allergic patients also had spirometry testing completed because they had some clinical history of respiratory symptoms. Two of the 15 had an obstructive pattern on their spirometry, as indicated by having a FEV1/FVC < 75% predicted. One patient was taking cetirizine and another was taking amitriptyline at the time of skin testing. These medications may have blunted the skin test response. Other common concomitant medications included HMG-CoA reductase inhibitors, diuretics, acetylsalicylic acid, angiotensin converting enzyme inhibitors, and calcium channel blockers.

**Table 1:** Characterization of Atopic Patients Assessed (N = 72)

		n (%)
Reason for referral		

	General allergy assessment	34 (47)

	Environmental allergies	13 (18)

	Rhinitis or sinusitis	7 (10)

	Anaphylactic event	5 (7)

	Rash or urticaria	5 (7)

	Food allergy	4 (6)

	Asthma	4 (6)

	Angioedema	3 (4)

	Cough	3 (4)

	Drug allergy	2 (3)

		

Spirometry performed		15 (21)

	FEV1% <80% predicted	3 (4)

	FEV1/FVC % <75% predicted	2 (13)

		

Other medications		

	HMG-CoA reductase inhibitor Iinhibitors inhibitors	22 (31)

	Diuretic	19 (26)

	ASA	18 (25)

	ACEi	14 (19)

	CCB	10 (14)

	Amitriptyline	1 (1)

	Cetirizine	1 (1)

ASA - acetyl salicylic acid, ACEi - angiotensin converting enzyme inhibitor, CCB - calcium channel blocker

The types of beta-blockers taken by the 72 patients with positive skin findings are summarized in table 2. The majority of patients were on oral agents, with atenolol and propanolol being the most commonly used. Two patients were taking ocular topical beta-blocking agents. Table 3 shows the indications for beta-blocker use. A few patients had more than one indication documented in their charts.

**Table 2:** Assessment of beta-blockers used by patients (N = 72)

		n (% patients)
Beta-blocker route		

	Oral	70 (97)

	Ocular	2 (3)

Beta-blocker name		

	Atenolol	42 (58)

	Metoprolol	15 (21)

	Nadolol	4 (6)

	Acebutalol	4 (6)

	Propanolol	2 (3)

	Bisprolol	2 (3)

	Timolol	1 (1)

	Labetolol	1 (1)

**Table 3:** Indications for beta-blocker use (N = 72)

	n (%)
HTN	49 (68)

CAD	17 (24)

MI	7 (10)

Headache	2 (3)

Glaucoma	2 (3)

Arrhythmia	2 (3)

Tremor	1 (1)

HTN = hypertension, CAD = coronary artery disease, MI = myocardial infarction

The allergens the 72 allergic patients tested positive to are listed in table 4. The majority of positive reactions were to environmental aeroallergens and insect venoms, but some patients were found to be allergic to latex, foods and penicillin.

**Table 4:** Positive skin tests (N = 72)

	Number positive (%)
Ragweed	25 (35)

Tree	24 (33)

Grass	24 (33)

Mold	22 (31)

Dust mite	22 (31)

Cat	15 (21)

Yellow jacket*	15 (21)

Wasp*	11 (15)

Yellow hornet*	10 (14)

White faced hornet*	9 (13)

Dog	7 (10)

Guinea pig	3 (4)

Seafood	3 (4)

Hamster	2 (3)

Fruit	2 (3)

Latex	2 (3)

Nut	2 (3)

Horse	1 (1)

Vegetable	1 (1)

Penicillin*	1 (1)

Honeybee*	1 (1)

*Intradermally tested

## Discussion

Beta-blockers are relatively contraindicated in both skin testing and immunotherapy for three reasons. They may: 1) worsen anaphylaxis severity; 2) make treatment of anaphylaxis more difficult; and 3) increase the incidence of anaphylaxis itself. First, in terms of anaphylaxis severity, beta-blockers can increase synthesis and release of anaphylactic mediators [13,14], as well as enhance responsiveness of organs to the mediators released [15-18]. In atopic subjects, total IgE production may increase. Those with allergic asthma already have excessive alpha-adrenergic reactivity and beta-blockade may further exacerbate this problem. In the treatment of anaphylaxis, beta-blockade has been shown to blunt the effect of epinephrine in animal and human models [19-22]. Finally, in case reports of patients with severe anaphylactic reactions, several patients were coincidently on beta-blocker medications [23-29].

While all of the issues above concerning beta-blockers and anaphylaxis are important, we argue that with allergy skin prick testing and intradermal testing with insect venoms and penicillin, the risk of anaphylaxis is so low that testing can be completed safely on most patients taking beta-blockers. Our study failed to show any adverse events in patients undergoing skin testing while taking beta-blockers. Because systemic reactions to skin testing are so uncommon, larger prospective studies comparing patients who are taking and not taking beta-blockers should be performed to prove any risk of beta-blocker use. It is important to note that fatalities from skin testing regardless of beta-blocker use are extremely rare. Through a national questionnaire study with members of the American Academy of Allergy and Immunology, seven fatalities are known: two occurring in 1964, four between 1980-1983, and one in a more recent report surveying the period between 1990-2001 [30-32]. None of these patients were known to be using beta-blockers. Furthermore, we could not find any published reports identifying any life-threatening or fatal reactions when patients taking beta-blockers were skin prick tested. In contrast, there are 88 reports from the American Academy of Allergy and Immunology over this time of allergen immunotherapy fatalities, with 3 of these patients taking beta-blockers [33-35]. Thus, while there may be an increased risk of anaphylaxis in patients on beta-blockers undergoing immunotherapy, the degree of risk is likely smaller in patients undergoing allergy skin testing. Of course, standard safety measures should still be available in the clinics where skin testing is being performed.

This study provides information regarding the safety of skin prick testing in patients taking beta-blocker medications. To date, this is the first study known which assesses this issue specifically. Our retrospective study showed no adverse effects of skin testing in 191 patients on beta-blockers. The fact that 72 patients with positive skin tests were able to tolerate the skin testing without any problems suggests skin testing is likely a safe procedure in most patients on beta-blockers. However, large multi-center prospective studies are required to truly measure any increased risk of anaphylaxis of allergy skin testing in patients taking beta-blockers. Until these larger studies are performed, skin testing should still be performed with caution in patients taking beta-blocker medications.

## Competing interests

The authors declare that they have no competing interests.

## Authors' contributions

IF helped to design the study, collected the data for analysis and wrote the paper. HK conceived the study and participated in its design and writing. All authors read and approved the final manuscript.
